# Risk of asthma in preterm infants with bronchopulmonary dysplasia: a systematic review and meta-analysis

**DOI:** 10.1007/s12519-023-00701-1

**Published:** 2023-03-01

**Authors:** Tong Sun, Hai-Yang Yu, Miao Yang, Yi-Fan Song, Jian-Hua Fu

**Affiliations:** 1 Department of Pediatrics, Shengjing Hospital of China Medical University, Sanhao Street, Heping District, Shenyang, China; 2 Department of Neurology, Shengjing Hospital of China Medical University, Shenyang, China

**Keywords:** Asthma, Bronchopulmonary dysplasia, Preterm infants

## Abstract

**Background:**

This study aimed to systematically review and meta-analyze the available literature on the association between preterm infant bronchopulmonary dysplasia (BPD) and pre-adulthood asthma.

**Methods:**

Studies examining the association between BPD and asthma in children and adolescents were systematically reviewed, and a meta-analysis was conducted. We searched Scopus, Embase, Web of Science, PubMed, and Cochrane Library from the database inception to March 26, 2022. The pooled odds ratio (OR) estimate was used in our meta-analysis to calculate the correlation between BPD and the probability of developing asthma before adulthood. Stata 12.0 was used to conduct the statistical analysis.

**Results:**

The correlation between asthma and BPD in preterm newborns was examined in nine studies. We used a random effect model to pool the OR estimate. Our results indicated a marked increase in the risk of subsequent asthma in preterm infants with BPD [OR = 1.73, 95% confidence interval (CI) = 1.43–2.09]. Moreover, there was no obvious heterogeneity across the studies (*P* = 0.617, *I*^2^ = 0%). The pooled OR remained stable and ranged from 1.65 (95% CI = 1.35–2.01) to 1.78 (95% CI = 1.43–2.21). Regarding publication bias, the funnel plot for asthma risk did not reveal any noticeable asymmetry. We further performed Begg’s and Egger’s tests to quantitatively evaluate publication bias. There was no evidence of a publication bias for asthma risk (*P* > |*Z*| = 0.602 for Begg’s test, and *P* > |*t*| = 0.991 for Egger’s test).

**Conclusions:**

Our findings indicate that preterm infants with BPD have a much higher risk of developing asthma in the future (OR = 1.73, 95% CI = 1.43–2.09). Preterm infants with BPD may benefit from long-term follow-up.

**Supplementary Information:**

The online version contains supplementary material available at 10.1007/s12519-023-00701-1.

## Introduction

Bronchopulmonary dysplasia (BPD) is a chronic lung disease and the most common complication affecting preterm infants. Additionally, BPD is complicated, with varying levels of severity and causes. The diagnosis of BPD is not always accurate and may vary depending on the area. Shennan et al. defined the diagnostic criterion as oxygen dependency at 36 weeks’ postmenstrual age (PMA) [1]. The National Institutes of Health (NIH) (2001 and 2018) defines the identification and classification of the severity of BPD based on the subjective requirements for various respiratory support modalities, which is a definition used more frequently. The NIH’s 2001 definition emphasized that infants require a fraction of inspired oxygen > 0.21 for no less than 28 days. In 2018, the NIH defined the diagnosis of BPD as infants ≤ 32 weeks gestational age with persistent radiographic findings of parenchymal lung disease who required respiratory support at 36 weeks PMA for ≥ 3 days to maintain arterial O_2_ saturation at 90%–95% [2, 3]. Although improvements in treatment have increased survival rates, the incidence of BPD either remained constant or even increased. Additionally, BPD affects not only the lungs but also the health and quality of life of preterm infants on a long-term basis [4].

BPD can impair pulmonary function, as shown by Vom Hove et al. in a study comparing adolescent or young adult BPD survivors with controls, with the former having reduced forced vital capacity (FVC), forced expiratory volume in 1 second (FEV_1_), and forced expiratory flow rate at 50% of FVC [5]. Pérez-Tarazona et al.’s study also showed that the FEV_1_, FVC, FEV_1_/FVC ratio, and forced expiratory flow between 25% and 75% of FVC were considerably lower in adolescents born extremely preterm with BPD than in other included adolescents [6]. Furthermore, BPD is also associated with the development of asthma, and many BPD survivors exhibit signs of reactive airway illness. More than double this number exhibited abnormal spirometry during long-term follow-up of preterm infants born at less than 26 weeks PMA, whereas 25% had an asthma diagnosis by 11 years of age [7]. A study by Di Fiore et al. found that BPD was strongly correlated with asthma after a two-year follow-up period [8]. In contrast, Astle et al. reported that the proportion of asthma was not significantly different between preterm infants with and without chronic lung disease [9].

Asthma, a multifactorial and heterogeneous chronic disease that commonly affects children, has had increasing global incidence over the past several decades, affecting almost 300 million individuals. It is defined by a history of respiratory symptoms, such as wheezing, shortness of breath, chest tightness, and cough, which vary over time and in intensity, and variable expiratory airflow limitations. The Global Initiative for Asthma’s diagnostic criteria for children (≥ 6 years old) include a clinical history that is suggestive of asthma, evidence of variability in expiratory airflow limitation on spirometry, which has bronchodilator reversibility, repeated peak expiratory flow measurements, positive exercise challenge, or a positive bronchial challenge test [10]. For children aged 1–5 years old, the diagnosis of asthma requires more than one presentation of asthma-like symptoms and a response to an asthma treatment trial [11]. The pathophysiology of asthma is influenced by a variety of host factors, genetic susceptibility, and environmental factors [12].

Early and accurate diagnosis of asthma in children is very difficult due to their inability to cooperate with some of the examinations and the underreporting of symptoms by patients or their caregivers. Underdiagnosis leads to failure to prescribe appropriate pharmaceutical and non-pharmaceutical therapies for asthma. Consequently, some children may be aggravated by untimely diagnosis, which will not only increase the poor prognosis but also cause a burden on the family and society. Therefore, it is important to study the risk factors associated with asthma. As the survival rate of preterm infants has improved, the incidence of BPD has also increased. Therefore, preterm infants with BPD may become one of the main contributing factors to the increasing population of children with asthma in the future. Therefore, it is very important to clarify the correlation between preterm infants with BPD and patients with asthma to increase the efficiency of asthma diagnosis and to provide early intervention and treatment in children with a medical history of BPD [13].

To date, insufficient data are available to evaluate BPD and the development of asthma, and most of the data are based on retrospective studies. It is, therefore, necessary to critically evaluate the current literature regarding the involvement of BPD in the development of asthma. The objective of this study was to estimate the association between BPD and asthma.

## Methods

This systematic review and meta-analysis were conducted in accordance with the Preferred Reporting Items for Systematic Reviews and Meta-Analyses (PRISMA) [14] and meta-analysis of observational studies in epidemiology guidelines [15]. The protocol for this review was registered in PROSPERO (ID: CRD42022323211) before the study screening.

### Literature search strategy

We searched PubMed, Scopus, Embase, Web of Science, and the Cochrane Library from database inception to March 26, 2022. The search strategy combined medical subject headings (MeSH) terms and free-text terms. The details of the full search strategy are described in Supplementary Table 1. Duplicate citations were removed. To identify any pertinent research, three independent authors (ST, YM, and SYF) reviewed the titles and abstracts. The full texts of eligible papers that seemed to satisfy the inclusion criteria were then acquired and evaluated independently by the three reviewers. All authors agreed to any differences after discussion.

### Inclusion and exclusion criteria

We included cross-sectional, cohort, or population-based case‒control studies that reported original data and (1) reported the diagnosis of asthma before adulthood in preterm infants with BPD; (2) had preterm infants without BPD in the comparison group; and (3) provided detailed information with a risk estimate and confidence interval. Reviews, letters, editorials, conference abstracts, studies conducted in languages other than English, and animal studies were excluded. Additionally, studies with the same population and those with populations over the age of 18 years were also excluded.

### Data extraction

We obtained the following data from every eligible study: publication year, first author’s name, geographic location, sample size, study design, gestational age, definitions and diagnosis of BPD, assessment and diagnosis of asthma, odds ratios (ORs) and 95% confidence intervals (CIs) for asthma associated with BPD, and the possible confounders considered or modified during the analysis.

### Study quality assessment

In the studies finally included in this review, the diagnosis of asthma was mainly determined by collecting medical history through patient questionnaires and supplementing medical history provided by parents/caretakers. Due to the inclusion time of the objectives, the diagnostic criteria for BPD were mostly based on the 2001 NIH definition.

We used the Newcastle‒Ottawa scale (NOS) to evaluate the studies’ quality (Review manager 5.2, Copenhagen: The Nordic Cochrane Center, The Cochrane Collaboration, 2012) [16]. The comparability of groups, selection of the study population, and identification of the exposure or result of interest were the three major criteria used to evaluate the quality of each observational study. The scale was based on a score out of nine, and a higher score was correlated with higher-quality articles.

### Statistical analysis

In this meta-analysis, we used the OR estimate to measure the association between BPD and the risk of developing subsequent asthma before adulthood. We used a random effect model to pool the OR estimates and obtain an overall estimate and the *Q* and *I*^2^ statistics to analyze the heterogeneity. If the *P* value was less than 0.10 or the *I*^2^ was greater than 50%, obvious heterogeneity might be present. To investigate the relationship between each study and the total pooled estimate, sensitivity analysis was performed. Egger’s test and Begg’s test were used to assess small study impacts such as publication bias. Statistical significance was defined as a two-tailed *P* value < 0.05. Statistical analysis was conducted using Stata 12.0 (StataCorp, College Station, Texas, USA).

## Results

### Literature search results

**Fig. 1:**
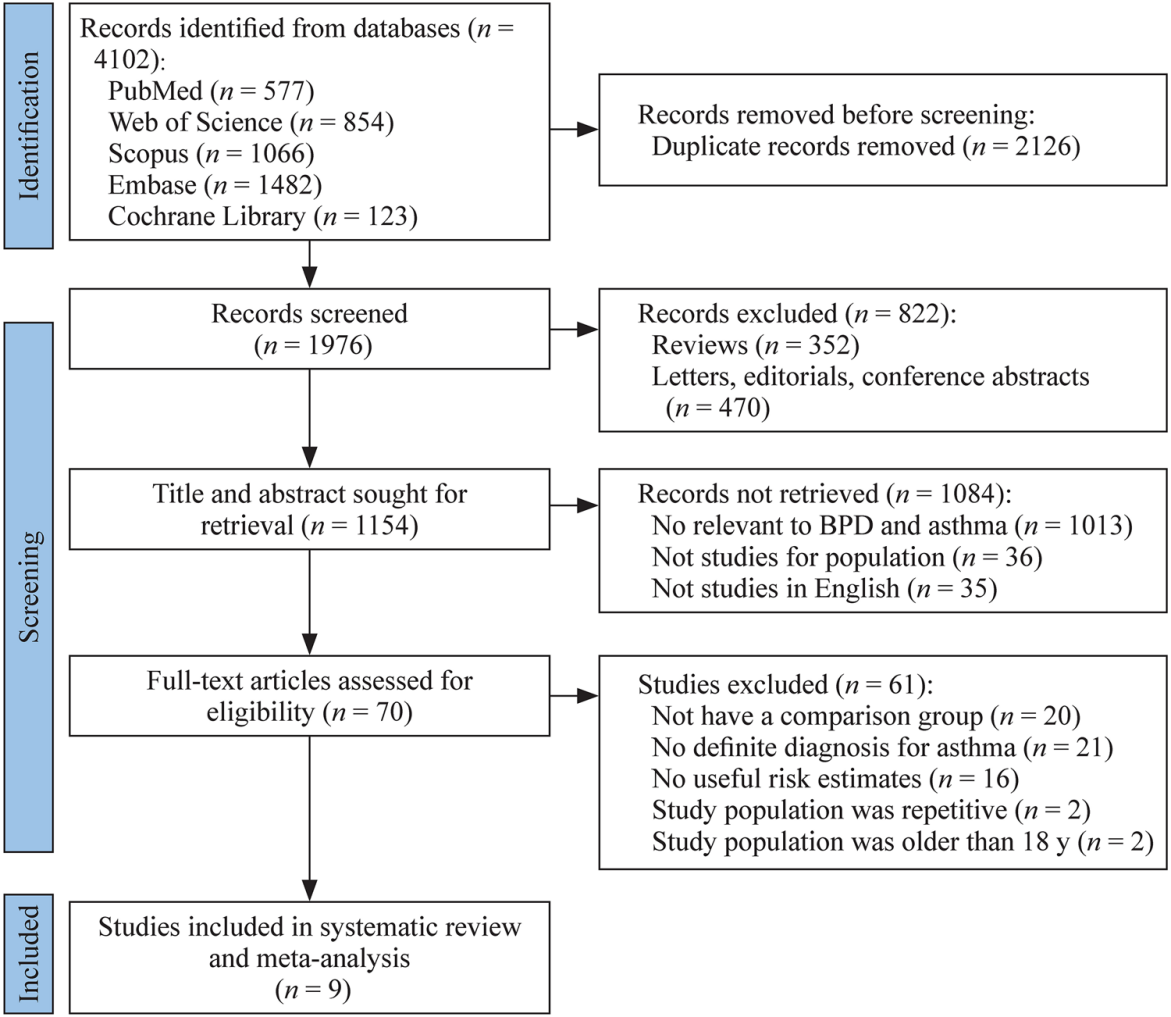
Flowchart of study selection included in this meta-analysis. *BPD* bronchopulmonary dysplasia

The PRISMA flowchart was used as a reference for the literature review and research selection procedure. We searched five databases and found 4102 records. After 2126 duplicate records and 822 other forms of literature were eliminated, 1154 records were retained for the title and abstract screening. A total of 1084 irrelevant records, animal studies, and studies written in languages other than English were excluded. The full texts of the remaining 70 studies were reviewed once more. Among them, 20 studies were excluded due to the lack of a comparison group, 21 studies were excluded because they had no definite diagnosis for asthma [17], 16 studies were eliminated due to the lack of any useful risk estimates or 95% CIs, two studies were excluded because the study population included patients over the age of 18 [18, 19], and two studies were excluded because they had the same study population [20, 21]. Nine full-text screening studies were thus included in this analysis (Fig. 1).

### Study characteristics and quality assessment

The characteristics and details of each study are presented in Table 1. Four studies were conducted in North America, two in Europe, two in Australia, and one in South America. Nine studies included 2279 preterm infants, 875 of whom developed BPD. Asthma assessment in these studies was mainly based on questionnaires. Age, height, body mass index, exposure to cigarette smoke, and pets in the home were among the most common confounders considered or adjusted for in most studies.

**Table 1:** Characteristics and details of the included studies

Study	Year	Study region	Study type	Participant characteristics	BPD diagnosis criteria	Follow-up age of asthma (y)	NOS scores
Pérez-Tarazona et al. [6]	2021	Spain	Cross-sectional	GA < 28 wk *n* = 184 (BPD: 92; non-BPD: 92)	NIH 2001 definition	Mean age: 14.2	7
Fierro et al. [22]	2019	America	Retrospective cohort study	GA < 30 wk *n* = 811 (BPD: 316; non-BPD: 495)	NIH 2001 definition	3–10	9
Doyle et al. [23]	2019	Australia	Cohort study	GA < 28 wk, or BW < 1000 g *n* = 297 (BPD: 121; non-BPD: 176)	Shennan 1988 definition	8	8
Di Fiore et al. [8]	2019	America	Retrospective cohort	GA < 28 wk *n* = 137 (BPD: 46; non-BPD: 91)	NIH 2001 definition	2	6
Skromme et al. [24]	2018	Norway	Prospective cohort	GA < 28 wk, or BW < 1000 g *n* = 372 (BPD: 165; non-BPD: 207)	NIH 2001 definition	< 11	9
Gonçalves et al. [25]	2016	Brazil	Cross-sectional	BW < 1500 g *n* = 54 (BPD: 18; non-BPD: 36)	NIH 2001 definition	9–10	6
Astle et al. [9]	2015	Australia	Cross-sectional	GA < 30 wk, or BW < 1000 g *n* = 53 (BPD: 28; non-BPD: 25)	Shennan 1988 definition	5–7	6
Grischkan et al. [26]	2004	America	Cohort study	GA < 36 wk *n* = 251 (BPD: 45; non-BPD: 206)	Shennan 1988 definition	8–11	7
Palta et al. [27]	2001	America	Cohort study	BW < 1500 g *n* = 120 (BPD: 44; non-BPD: 76)	Scale defined by the authors, including clinical and radiological criteria	8	8

*BPD* bronchopulmonary dysplasia, *NOS* Newcastle–Ottawa scale, *GA* gestational age, *BW* birth weight, *NIH* National Institutes of Health

The NOS scores of the study quality assessments were also calculated. Each study received a total score ranging from 6 to 9. Six of them had a score of no less than 7, indicating that they were extremely high-quality studies with a low risk of bias (Supplementary Fig. 1).

### Risk of asthma in preterm infants with bronchopulmonary dysplasia

**Fig. 2:**
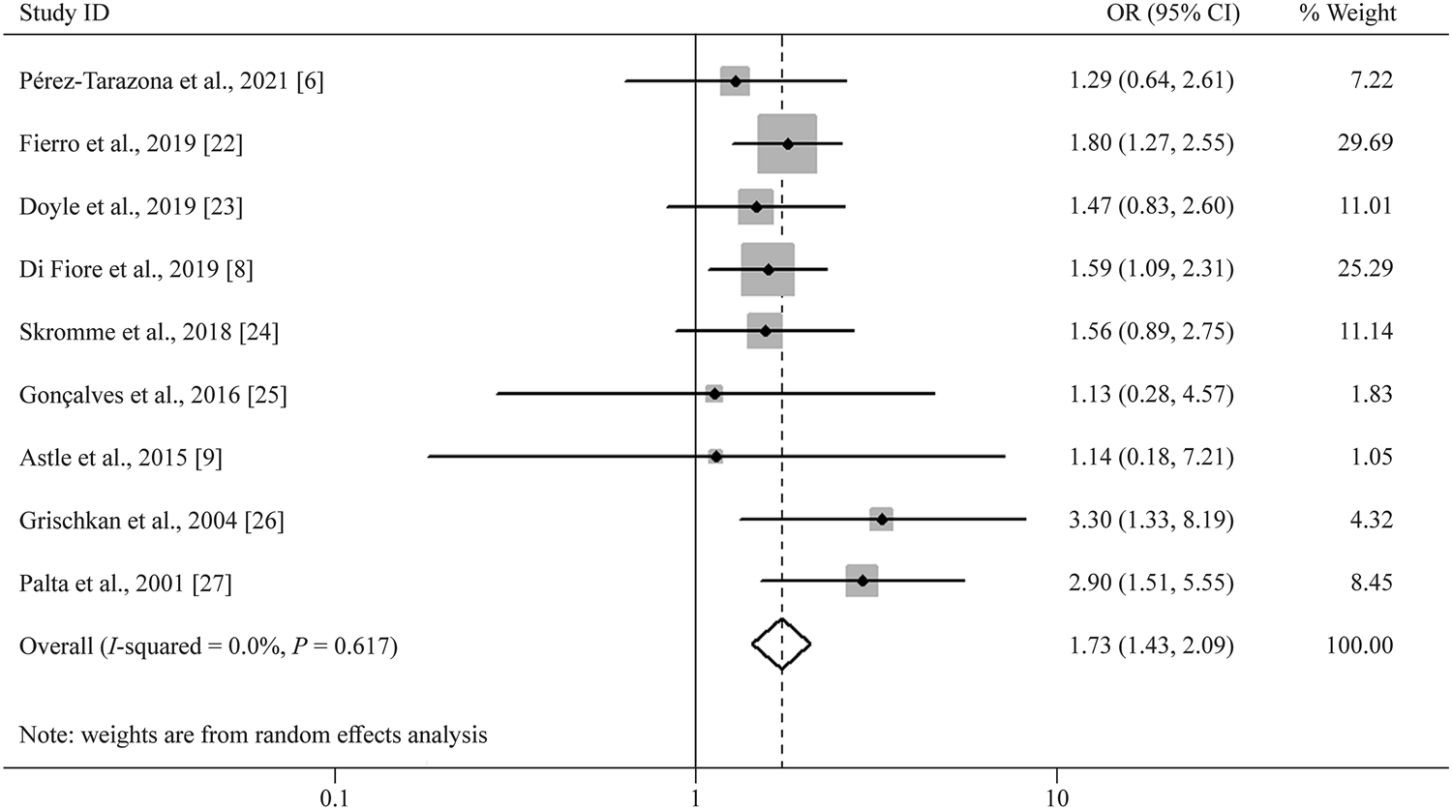
Forest plot for the relationship between bronchopulmonary dysplasia and the risk of asthma (OR = 1.73, 95% CI = 1.43–2.09, *I*^2^ = 0%, *P* = 0.617). A random effect model was used to create analytical weights. Gray boxes indicate study estimates, of which the size corresponds to the respective analytical weight. The 95% CIs for each study are represented by lines in the boxes. The pooled estimate is shown as a vertical black dashed line, and its 95% CI is shown as a diamond. *OR* odds ratio, *CI* confidence interval

Nine studies reported a relationship between asthma before adulthood and BPD in preterm infants. A random effect model was used to pool the effect estimate. The forest plot showed a significant increase in the risk of developing subsequent asthma in preterm infants with BPD (OR = 1.73, 95% CI = 1.43–2.09, Fig. 2). Moreover, there was no obvious heterogeneity across the studies (*P* = 0.617, *I*^2^ = 0%).

### Sensitivity analysis and publication bias

**Fig. 3:**
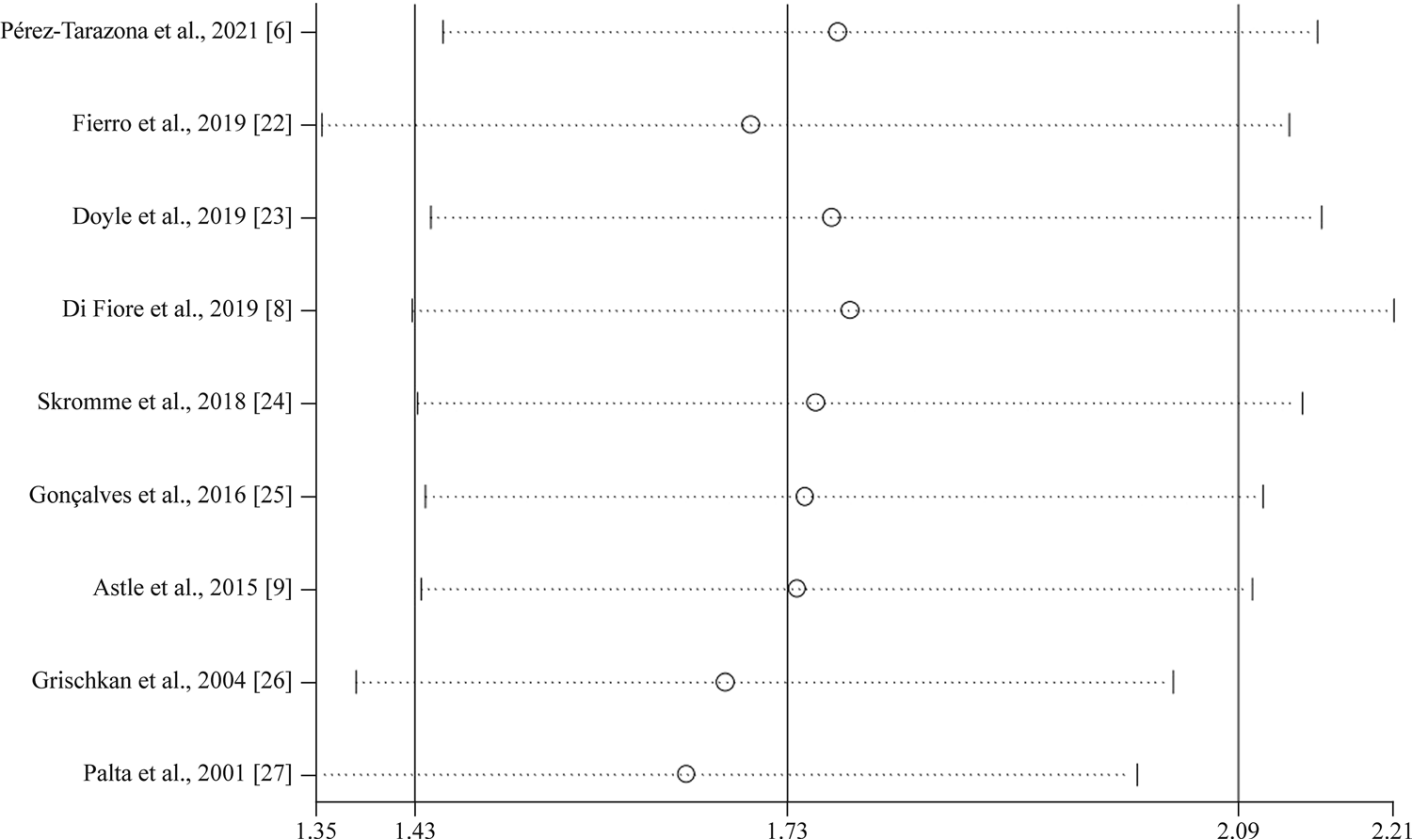
Sensitivity analysis for random-effect estimates (exponential form) with each study omitted

**Fig. 4:**
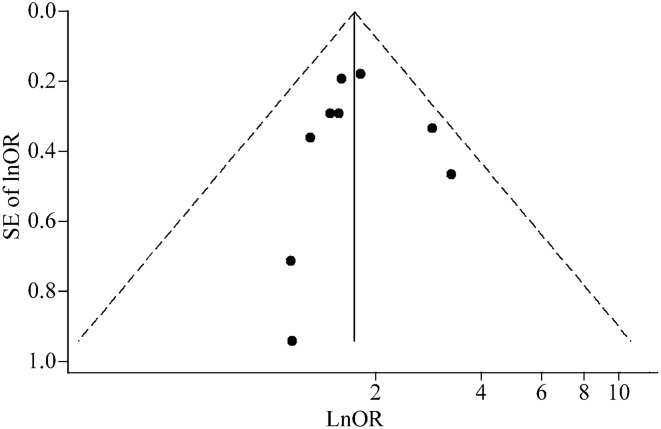
Funnel plot with pseudo 95% confidence limits. *SE* standard error, *OR* odds ratio

In terms of the sensitivity analyses, one study at a time was gradually eliminated, and the pooled ORs of the residual studies were recalculated. After the studies by Palta et al. [27] and Di Fiore et al. [8] were excluded, the pooled ORs did not change significantly and ranged from 1.65 (95% CI = 1.35–2.01) to 1.78 (95% CI = 1.43–2.21, Fig. 3). In terms of publication bias, the funnel plot for asthma risk showed some degree of asymmetry. However, we conducted Begg’s and Egger’s tests to quantitatively evaluate publication bias. The risk of asthma was not affected by publication bias (*P* > |*Z*| = 0. 602 for Begg’s test, and *P* > |*t*| = 0.991 for Egger’s test, Fig. 4).

## Discussion

According to the reviewed studies, preterm infants with BPD had a higher incidence of asthma than those without BPD. The conclusion is similar to that of Pérez-Tarazona’s [6] systematic review, even though a meta-analysis was not conducted. To the best of our knowledge, this is the first meta-analysis assessing the correlation between BPD and asthma before adulthood.

There is a debate about whether the respiratory symptoms experienced before adolescence by children with a history of BPD should qualify as asthma or if the two conditions, BPD and asthma, should be considered distinct illnesses. Although the two diseases have traits such as an inflammatory foundation, bronchial hyperresponsiveness, and persistent airflow restriction [28, 29] in common, additional distinctions should be considered. In contrast to BPD, which is characterized by a predominance of neutrophils and macrophages and a cytokine profile that is characteristic of a T helper (Th) 1-mediated response, asthmatic inflammation is characterized by mast cells and eosinophils, which exhibit a cytokine composition typical of Th2-mediated reactions. Additionally, BPD differs from atopic asthma in that it shows larger deficits in airflow and diffusion capacity as well as less persistent indications of airway inflammation [30].

Research on the mechanisms of asthma development in preterm infants with BPD is relatively limited. Impairment of lung function in preterm infants with BPD, which is longer in duration in infants with moderate-to-severe BPD, may persist until adulthood and be related to the occurrence of asthma [31]. Additionally, both BPD and asthma have similar respiratory symptoms, such as bronchial hyperresponsiveness [32]. Low birth weight and preterm birth may also be risk factors for BPD and asthma [33]. At the molecular level, Gao et al. reported that functional enrichments and pathways showed that the different expression of genes in BPD and non-BPD infants was mainly enriched in asthma [34]. Surfactant protein D, a pattern-recognition molecule belonging to the collectin family, was reported to correlate with the development of respiratory diseases, including allergic asthma and BPD [35]. Overactivation of pulmonary neuroendocrine cells, a kind of multi-functional epithelial cell that has an immune-regulatory function, has been reported in patients with asthma and BPD [36].

This study’s limitations included variations in the diagnostic criteria throughout the time period covered by our analysis, which are partially responsible for the diversity in the definition of BPD. Additionally, although there were differences in the diagnostic criteria for BPD and the method of diagnosis of asthma among the studies included in this review, heterogeneity analysis revealed no heterogeneity in the results of this study.

Although the included studies showed that there were no evident differences in age, height, living environment, and other factors in the study population, the influence of repeated viral infection after birth, family allergy, and other factors on the occurrence of asthma was still not entirely excluded. Only a small number of studies accurately assessed and controlled for the differences between patients with and without BPD with regard to the primary confounding variables. Future studies should include larger sample sizes to provide more robust conclusions.

In conclusion, this systematic review and meta-analysis found that preterm infants with BPD have a much higher chance of developing asthma later in life before adulthood (OR = 1.73, 95% CI = 1.43–2.09). Prospective studies utilizing uniform diagnostic standards for BPD and asthma would be more appropriate to accurately establish the potential link, considering the requirement for adequate assessment and adjustment for confounding variables. As preterm infants with BPD may eventually acquire asthma and develop the chronic obstructive pulmonary disease in their adult life, long-term follow-up is advised.

## Supplementary Information

Below is the link to the electronic supplementary material.

Supplementary file 1 (DOCX 227 KB)

## Data Availability

All data generated or analyzed during this study are included in this published article and its supplementary information files.
