# Supplementary material for: Risk of asthma in preterm infants with bronchopulmonary dysplasia: a systematic review and meta-analysis
Source: World J Pediatr. 2023 Mar 1;19(6):549–56. doi: 10.1007/s12519-023-00701-1 (PMC10198915; doi:10.1007/s12519-023-00701-1)
Supplement: Supplementary file 1 — (DOCX 227 KB) [file 12519_2023_701_MOESM1_ESM.docx]

**Supplementary Table 1.** Literature search strategy

| Up to March 26, 2022 |
| --- |
| **PubMed: all fields** |
| #3: #1 AND #2 |
| (((bronchopulmonary dysplasia) OR (lung dysplasia)) OR (BPD)) AND (((((asthma) OR (asthmas)) OR (bronchial asthma)) OR(wheeze)) OR(wheezing)) |
| #2: ((((asthma) OR(asthmas)) OR (bronchial asthma)) OR (wheeze)) OR (wheezing) |
| #1: ((bronchopulmonary dysplasia) OR (lung dysplasia)) OR (BPD) |
| **Web of Science: TS** |
| TS = (bronchopulmonary dysplasia OR lung dysplasia OR BPD) AND TS = (asthma OR asthmas OR bronchial asthma OR wheeze OR wheezing) |
| **Cochrane Library: all text** |
| (((bronchopulmonary dysplasia) OR (lung dysplasia)) OR (BPD)) AND (((((asthma) OR (asthmas)) OR (bronchial asthma)) OR (wheeze)) OR (wheezing)) |
| **Embase: all fields** |
| #3: #1 AND #2 |
| #2: “asthma”/exp OR asthma OR asthmas OR (bronchial AND asthma) OR wheeze OR wheezing |
| #1: “bronchopulmonary dysplasia”/exp OR “bronchopulmonary dysplasia” OR (bronchopulmonary AND (“dysplasia”/exp OR dysplasia)) OR (lung AND dysplasia) OR bpd |
| **Scopus: TITLE-ABS-KEY** |
| ((TITLE-ABS-KEY (bronchopulmonary AND dysplasia) OR TITLE-ABS-KEY (lung AND dysplasia) OR TITLE-ABS-KEY (bpd))) AND ((TITLE-ABS-KEY (asthma) OR TITLE-ABS-KEY (asthmas) OR TITLE-ABS-KEY (bronchial AND asthma) OR TITLE-ABS-KEY (wheeze) OR TITLE-ABS-KEY (wheezing))) |

**
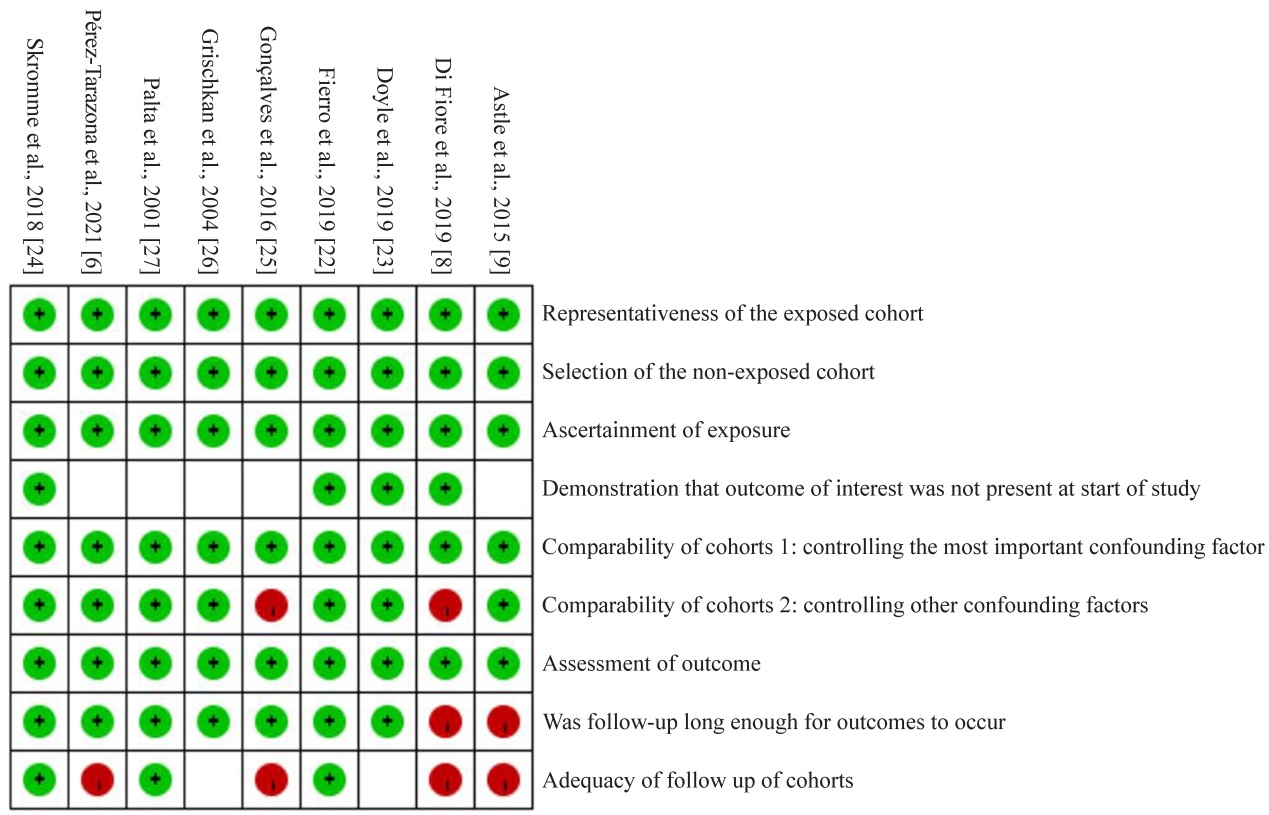
**

**Supplementary Fig. 1** Newcastle-Ottawa Scale scores of study quality for included studies. Green circle means 1 point, red circle or blank means 0 point. The full points are 9
